# Regenerative therapies for femoral head necrosis in the past two decades: a systematic review and network meta-analysis

**DOI:** 10.1186/s13287-024-03635-1

**Published:** 2024-01-25

**Authors:** Xiaole Wang, Liyou Hu, Bo Wei, Jian Wang, Decai Hou, Xiaolei Deng

**Affiliations:** 1 Affiliated Hospital of Shandong University of Traditional Chinese Medicine, Jingshi Road 16369, Jinan, 250014 China; 2 Liaoning University of Traditional Chinese Medicine, Chongshan Road 79, Shenyang, 110032 China; 3 Affiliated Hospital of Liaoning University of Traditional Chinese Medicine, Beiling Street 33, Shenyang, 110032 China

**Keywords:** Regenerative therapies, Stem cells, Bone marrow aspirate concentrate, Femoral head necrosis, Bayesian network meta-analysis

## Abstract

**Background:**

Regenerative techniques combined with core decompression (CD) are commonly used to treat osteonecrosis of the femoral head (ONFH). However, no consensus exists on regeneration therapy combined with CD that performs optimally. Therefore, we evaluated six regenerative therapies combined with CD treatment using a Bayesian network meta-analysis (NMA).

**Methods:**

We searched PubMed, Embase, Cochrane Library, and Web of Science databases. Six common regeneration techniques were categorized into the following groups with CD as the control group: (1) autologous bone graft (ABG), (2) autologous bone graft combined with bone marrow aspirate concentrate (ABG + BMAC), (3) bone marrow aspirate concentrate (BMAC), (4) free vascular autologous bone graft (FVBG), (5) expanded mesenchymal stem cells (MSCs), and (6) platelet-rich plasma (PRP). The conversion rate to total hip arthroplasty (THA) and progression rate to femoral head necrosis were compared among the six treatments.

**Result:**

A total of 17 literature were included in this study. In the NMA, two of the six treatment strategies demonstrated higher response in preventing the progression of ONFH than CD: MSCs (odds ratio [OR]: 0.098, 95% confidence interval [CI]: 0.0087–0.87) and BMAC (OR: 0.27, 95% CI: 0.073–0.73). Additionally, two of the six treatment strategies were effective techniques in preventing the conversion of ONFH to THA: MSCs (OR: 0.062, 95% CI: 0.0038–0.40) and BMAC (OR: 0.32, 95% CI: 0.1–0.074). No significant difference was found among FVBG, PRP, ABG + BMAC, ABG, and CD in preventing ONFH progression and conversion to THA (*P* > 0.05).

**Conclusions:**

Our NMA found that MSCs and BMAC were effective in preventing ONFH progression and conversion to THA among the six regenerative therapies. According to the surface under the cumulative ranking value, MSCs ranked first, followed by BMAC. Additionally, based on our NMA results, MSCs and BMAC following CD may be necessary to prevent ONFH progression and conversion to THA. Therefore, these findings provide evidence for the use of regenerative therapy for ONFH.

**Supplementary Information:**

The online version contains supplementary material available at 10.1186/s13287-024-03635-1.

## Background

Osteonecrosis of the femoral head (ONFH) is a common refractory disease in joint orthopedics. More than 10,000 new patients are affected with ONFH annually in the United States, accounting for approximately 10% of total hip arthroplasties (THAs) [1]. The cumulative number of patients with ONFH in China reached 8.12 million in 2013 [2]. According to statistics, the prevalence rate of ONFH is increasing yearly [3]. ONFH is a progressive disease typically caused by insufficient blood supply to the femoral head, which leads to increased pressure in it, eventually culminating in its collapse. The femoral head usually develops into secondary arthritis when it collapses [4]. Core decompression (CD) is a commonly used procedure for treating femoral head necrosis despite some controversy; it is a simple procedure that treats ONFH by drilling into the necrotic area of the femoral head [5–8]. The theoretical advantage of CD is in relieving the pain by reducing venous congestion and bone marrow pressure. Blood flow increases in the osteonecrosis area with the decrease in intraosseous pressure, thereby alleviating the pathology and promoting bone regeneration in the osteonecrosis area [9, 10]. CD combined with regeneration therapy appears to accelerate the healing of osteonecrosis and reduce the risk of femoral head collapse [11]. Recently, studies have shown that bone marrow aspirate concentrate (BMAC), expanded mesenchymal stem cells (MSCs), autologous bone graft (ABG), and other regenerative therapies show gratifying outcomes in the treatment of bone diseases [12–17]. In addition to BMAC, MSCs, and ABG, common regeneration therapies also include platelet-rich plasma (PRP), autologous bone graft combined with bone marrow aspirate concentrate (ABG + BMAC), and free vascular autologous bone graft (FVBG). Despite the promising results of these diferent methods, the best regeneration therapy for ONFH has not yet been determined.

Bayesian network meta-analysis (NMA), also known as multiple treatment comparison meta-analysis, can simultaneously analyze direct and indirect evidence from different studies, expand the scope of traditional conventional pairwise analysis, and subsequently estimate the relative effectiveness of all interventions and rank them [18]. To date, no comparison of the different regenerative therapies has been performed for ONFH using NMA. Herein, we used a Bayesian NMA to evaluate the efficacy of different regenerative therapies based on ONFH progression and conversion to THA.

## Methods

This systematic review and NMA adhered to the guidelines outlined in the Preferred Reporting Items for Systematic Reviews and Meta-Analyses (PRISMA) statement [19]. Additionally, our review was registered on PROSPERO (http://www.crd.york.ac.uk/PROSPERO) under the registration number CRD42023412784.

### Search strategy

All articles published between 2003 and 2023 in PubMed, EMBASE, Cochrane Library, and Web of Science databases were searched. We used the following keywords: “femur head” AND (“bone necrosis” OR “avascular necrosis” OR “osteonecrosis”) AND (“regenerative therapies” OR “stem cells” OR “bone marrow” OR “bone graft” OR “platelet rich plasma”). An additional file presents the details of the search process (see Additional file 1).

### Study selection

The inclusion and exclusion process followed the PICOS (Participants, Intervention, Comparison, Outcome, and Study) principle. Additionally, the mean age of patients with ONFH was 18 years. Studies include at least two of the following treatments: ABG, ABG + BMAC, BMAC, CD, MSCs, FVBG, and PRP. The included studies reported at least one of the two outcomes as follows: the rate of THA requirement and that of ONFH stage progression after the intervention. Furthermore, the included studies were randomized controlled trials (RCTs) or retrospective cohort studies conducted in English, published from 2003 to 2023.

The exclusion criteria were as follows: Non-English text; literature with a low-quality treatment evaluation; and reviews, protocols, case reports, conference papers, and animal experiments.

All relevant studies were screened independently by two reviewers, and any disagreement between the two reviewers regarding a study’s eligibility was resolved through discussion with a third reviewer.

### Data extraction

Two independent reviewers extracted the following information from each included study: the first author’s surname, year of publication, study types, follow-up time, average age, hip sample size, ONFH staging of patient, conversion to THA, and ONFH progression. Any differences were resolved through discussion with a third reviewer.

### Quality assessment

Two independent reviewers assessed the literature quality. RCT and retrospective cohort studies were assessed for quality using the Cochrane Risk of Bias tool [20] and the Newcastle–Ottawa Scale (NOS), respectively. The following factors were assessed for each study: randomization sequence generation (selection bias), allocation concealment, subject blinding, outcome assessment, attrition bias (incomplete outcome data), reporting bias (selective reporting), and other biases. Studies with scores of 8 and 9, and 6 and 7 were considered high and medium quality studies, respectively [21]. Any differences were resolved through discussion with a third reviewer.

### Statistical analysis

We analyzed the following two metrics: ONFH conversion rates to THA and its progression rates. The results are expressed as the odds ratio (OR) and 95% confidence interval (CI). A pairwise meta-analysis was performed using R software (version 5.35; Lucent Technologies, Paris, France).

Heterogeneity between comparable studies was examined using the chi-square (*χ*^2^) and *I*^2^ tests. Values < 25%, 25–75%, and > 75% for the I^2^ statistic represented mild, moderate, and severe heterogeneity, respectively [22]. Furthermore, node-splitting analysis was used to assess the inconsistency of a particular comparison based on direct and indirect evidence; statistical significance was considered at P < 0.05 [23]. Furthermore, funnel plots were used to test for publication bias.

We also calculated the surface under the cumulative ranking (SUCRA) value, a simple numerical summary to supplement the graphical display of cumulative ranking, which is used to estimate the SUCRA line for each treatment. The SUCRA values of 1 and 0 signify a treatment that is certain to be optimal and the worst, respectively [23].

## Results

### Study selection and characteristics

**Fig. 1:**
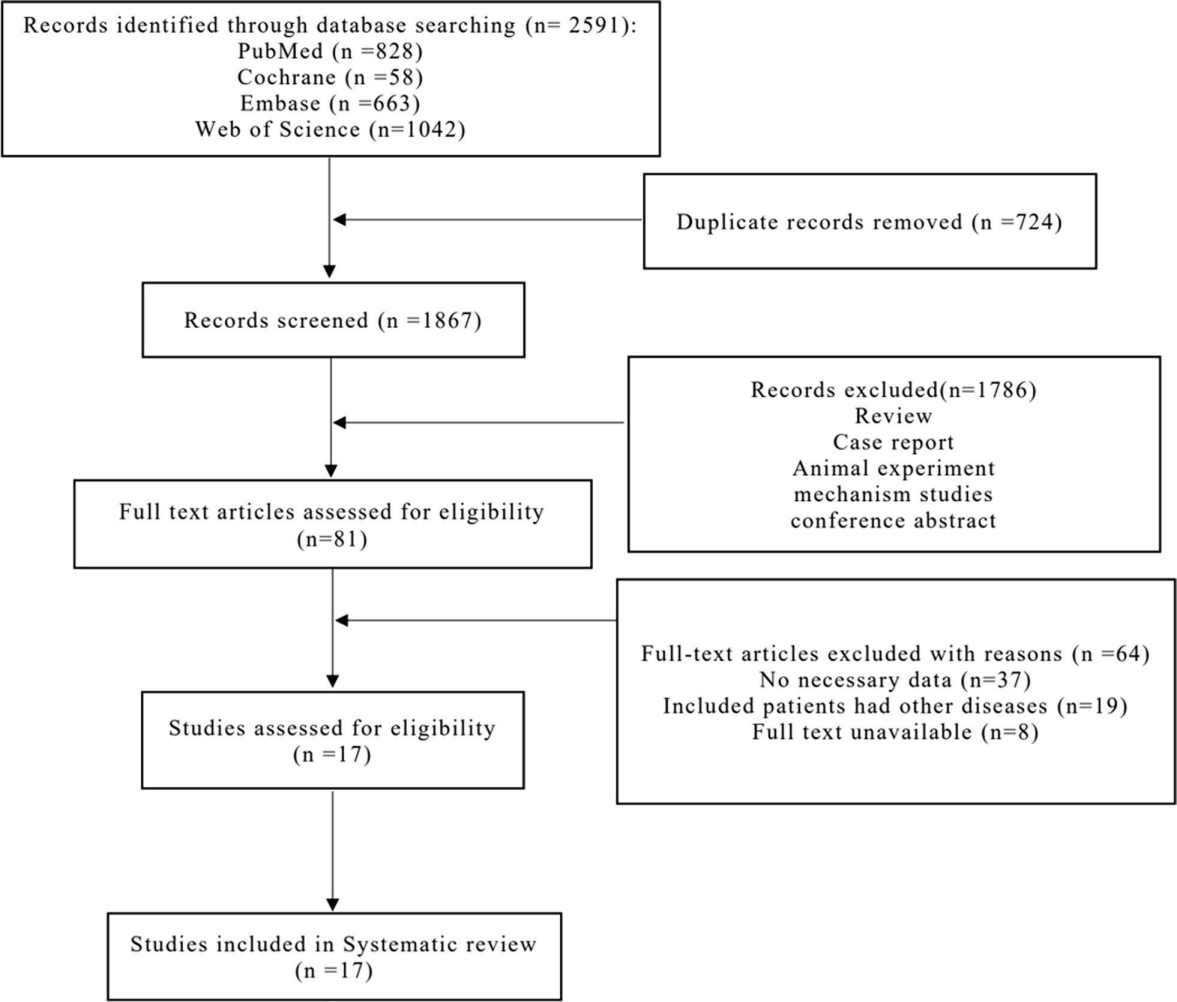
Flowchart of study selection and design

**Fig. 2:**
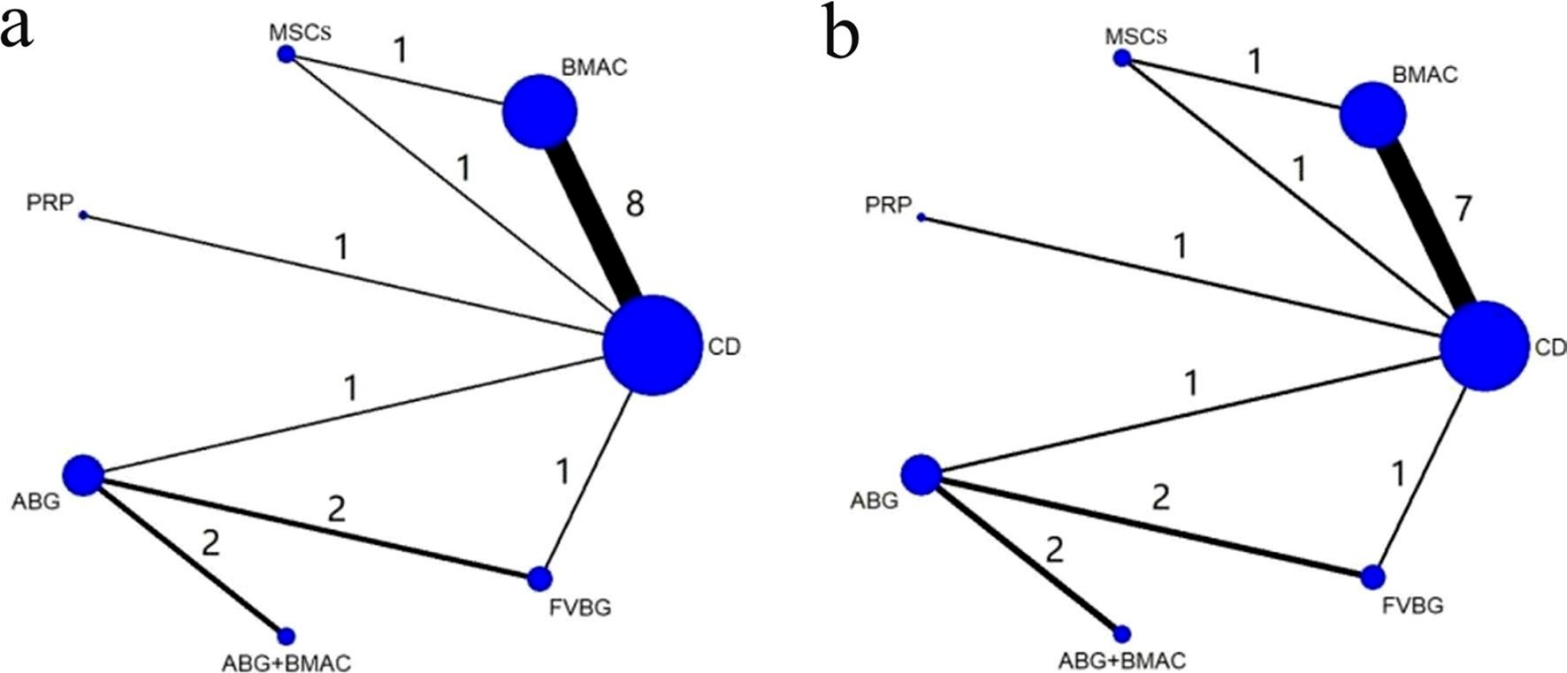
Network plots of comparison-based network meta-analyses. Each circular node represents a type of intervention. The circle size is proportional to the total number of patients. The width of the lines is proportional to the number of studies performing head-to-head comparisons in the same study. **a** ONFH progression and **b** conversion to total hip arthroplasty (THA). ABG: autologous bone grafting; ABG + BMAC: autologous bone grafting and bone marrow aspirate concentrate; BMAC: bone marrow aspirate concentration; CD: core decompression; FVBG: free vascular vascularized bone grafting; MSCs: mesenchymal stem cells; PRP: platelet-rich plasma

Figure 1 shows the study selection process. We retrieved 2591 articles, ultimately including 17 studies. A total of 1019 hips were included in our NMA groups, comprising 245, 80, 25, 177, 50, 151, and 291 hips in the BMAC, MSCs, PRP, ABG, ABG + BMAC, FVBG, and CD groups, respectively. Table 1 presents the basic characteristics of the included studies. The network structure of the analyzed comparisons for the primary outcomes is shown in Fig. 2.

**Table 1:** Characteristics of included individual studies

Author	Year	Country	Design	Treatment	Mean age (years)	Case (hip)	Inclusion criteria	Follow-up (years)	Outcomes
Gangji [24]	2011	Belgium	RCT	CD BMAC	45.7 42.2	11 13	ARCO I-II	5	THA, Progress
Sen [25]	2012	India	RCT	CD BMAC	31.1 34.7	25 26	ARCO I-II	2	Progress
Zhao [26]	2012	China	RCT	CD MSCs	33.8 32.7	44 53	ARCO I-II	5	THA, Progress
Rastogi [27]	2013	India	RCT	CD BMAC	33 34.7	30 30	ARCO I-III	2	THA, Progress
Ma [28]	2014	China	RCT	ABG ABG + BMAC	34.8 35.6	24 25	Ficat I-III	2	THA, Progress
Tabatabaee [29]	2015	Iran	RCT	CD BMAC	29.1 29.1	14 14	ARCO I-III	2	THA, Progress
Pardos [30]	2016	Spain	Retrospective cohort	CD BMAC	36.7 42.6	19 41	Ficat I-II	4	THA, Progress
Pepke [31]	2016	Germany	RCT	CD BMAC	44.5 44.3	14 11	ARCO II	2	THA, Progress
Sallam [32]	2017	Egypt	Retrospective cohort	CD ABG	33.2 32.6	38 33	Ficat I-III	3	THA, Progress
Hauzeur [33]	2017	Belgium	RCT	CD BMAC	49.7 48	23 23	ARCO III	2	THA, Progress
Cao [34]	2017	China	RCT	CD FVBG	31 31	21 21	ARCO I-III	3	THA, Progress
Feng [35]	2019	China	Retrospective cohort	FVBG ABG	33.2 32.8	84 51	ARCO III	6	THA, Progress
Hauzeur [36]	2019	Belgium	RCT	BMAC MSCs	50 51	26 27	ARCO I-II	3	THA, Progress
Aggarwal [37]	2020	India	RCT	CD PRP	35.2 38.2	28 25	ARCO I-II	1	THA, Progress
Li [17]	2020	China	RCT	ABG ABG + BMAC	38.2 34.1	24 25	Ficat I-III	2	THA, Progress
Hoogervorst [38]	2022	America	Retrospective cohort	CD BMAC	39.8 33.1	24 61	ARCO I-IV	5	THA, Progress
Wan [39]	2022	China	RCT	ABG FVBG	29.6 28.8	45 46	ARCO II	4	THA, Progress

*ABG* autologous bone grafting, *ABG* + *BMAC* autologous bone grafting and bone marrow aspirate concentrate, *ARCO* Association Research Circulation Osseous, *BMAC* bone marrow aspirate concentration, *CI* confidence interval, *CD* core decompression, *FVBG* free vascular vascularized bone grafting, *MD* mean difference, *MSCs* mesenchymal stem cells, *NMA* network meta-analysis, *OR* odds ratio, *ONFH* osteonecrosis of the femoral head, *PRP* platelet-rich plasma, *RCT* randomized controlled trial, *THA* total hip arthroplasty

**Fig. 3:**
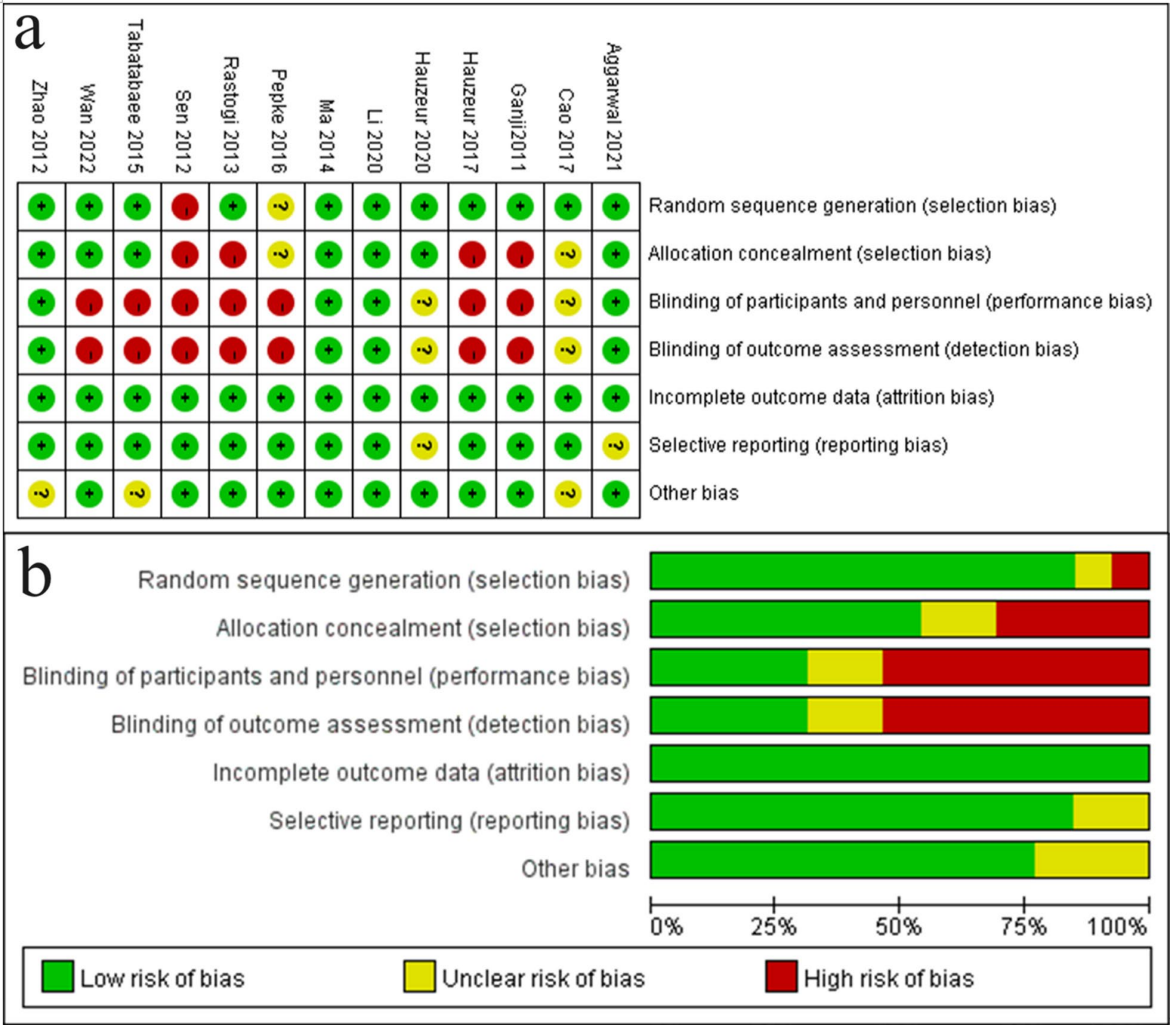
Assessing the quality of randomized control trials (RCTs) using the Cochrane Risk of Bias tool, **a**: Risk of bias graph. **b**: Summary of study risk bias analysis

**Fig. 4:**
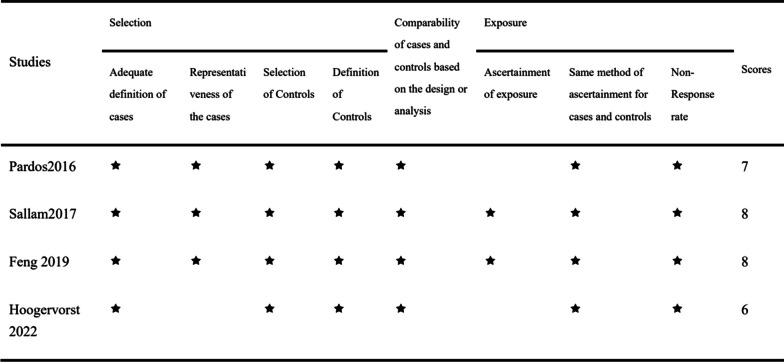
Assessing the quality of retrospective cohort studies using the Newcastle–Ottawa Scale

**Fig. 5:**
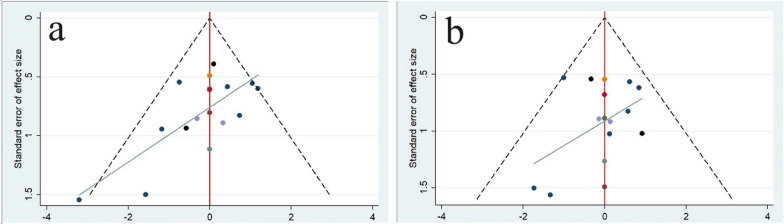
Funnel diagram of the included studies. **a** Osteonecrosis of the femoral head (ONFH) progression and **b** conversion to total hip arthroplasty (THA)

For RCTs using the Cochrane Risk of Bias tool, the overall quality assessment showed a low or moderate risk of bias, with a higher risk observed in the blinded component, mainly because the procedure required informed consent and it was difficult for the operator and patient to be blinded; however, this does not imply that the study was meaningless (Fig. 3). Retrospective cohort studies were assessed using the NOS, revealing two medium-quality (score: 6 or 7) and two high-quality (score: 8 or 9) studies (Fig. 4). We assessed a funnel diagram of the included studies (Fig. 5), and the roughly symmetrical diagram suggests no publication bias.

Heterogeneity was found among the comparisons of treatments on the rates of ONFH progression and conversion to THA (Tables 2, 3). We found that the Bayesian NMA results were reliable; the inconsistency between the direct and indirect effects of comparisons of different treatments on the two outcomes showed no significant differences (Tables 4, 5). The nodal split method for ONFH progression and conversion to THA showed no significant heterogeneity (Tables 6, 7).

**Table 2:** Heterogeneity results of ONFH progression according to pairwise meta-analysis

Comparison	Number of studies included	Progress at last follow-up time point
		I^2^
CD vs. BMAC	8	46.97%
CD vs. MSCs	1	–
CD vs. PRP	1	–
CD vs. ABG	1	–
CD vs FVBG	1	–
BMAC vs MSCs	1	–
ABG vs FVBG	2	0.00%
ABG vs ABG + BMAC	2	0.00%

*ABG* autologous bone grafting, *ABG* + *BMAC* autologous bone grafting and bone marrow aspirate concentrate, *BMAC* bone marrow aspirate concentration, *CD* core decompression, *FVBG* free vascular vascularized bone grafting, *MSCs* mesenchymal stem cells, *ONFH* osteonecrosis of the femoral head, *PRP* platelet-rich plasma

**Table 3:** Heterogeneity results of conversion to THA according to pairwise meta-analysis

Comparison	Number of studies included	Conversion to THA at last follow-up time point
		*I*^2^
CD vs. BMAC	7	28.28%
CD vs. MSCs	1	–
CD vs. PRP	1	–
CD vs. ABG	1	–
CD vs FVBG	1	–
BMAC vs MSCs	1	–
ABG vs FVBG	2	14.29%
ABG vs ABG + BMAC	2	0.00%

*ABG* autologous bone grafting, *ABG* + *BMAC* autologous bone grafting and bone marrow aspirate concentrate, *BMAC* bone marrow aspirate concentration, *CD* core decompression, *FVBG* free vascular vascularized bone grafting, *MSCs* mesenchymal stem cells, *PRP* platelet-rich plasma, *THA* total hip arthroplasty

**Table 4:** Odds ratios of osteonecrosis of the femoral head progress between treatment groups

ABG	4.88 (0.49, 53.74)	0.76 (0.06, 11.5)	2.09 (0.08, 58.82)	0.21 (0.02, 2.05)	1.45 (0.22, 11.56)	0.51 (0.01, 22.72)
	ABG + BMAC	0.15 (0, 5.7)	0.42 (0.01, 25.09)	0.04 (0.01, 1.1)	0.3 (0.01, 6.71)	0.1 (0, 8.69)
		BMAC	2.77 (0.27, 25.46)	0.27 (0.07, 0.72)	1.92 (0.12, 32.05)	0.68 (0.02, 14.97)
			MSCs	0.1 (0.01, 0.87)	0.69 (0.02, 23.25)	0.24 (0, 10.04)
				CD	7.08 (0.68, 111.62)	2.49 (0.12, 53.5)
					FVBG	0.35 (0.01, 15.24)
						PRP

*ABG* autologous bone grafting, *ABG* + *BMAC* autologous bone grafting and bone marrow aspirate concentrate, *BMAC* bone marrow aspirate concentration, *CD* core decompression, *FVBG* free vascular vascularized bone grafting, *MSCs* mesenchymal stem cells, *PRP* platelet-rich plasma

**Table 5:** Odds ratios of conversion to total hip arthroplasty between treatment groups

ABG	2.88 (0.46, 20.88)	1.79 (0.24, 18.33)	9.42 (0.7, 297.19)	0.58 (0.09, 3.92)	2.25 (0.42, 10.57)	1.7 (0.07, 50.38)
	ABG + BMAC	0.62 (0.04, 12.36)	3.25 (0.13, 166.78)	0.2 (0.01, 2.85)	0.78 (0.06, 8.23)	0.58 (0.01, 26.96)
		BMAC	5.21 (0.83, 60.65)	0.32 (0.1, 0.74)	1.24 (0.09, 11.31)	0.94 (0.05, 15.9)
			MSCs	0.06 (0.001, 0.4)	0.24 (0.01, 3.71)	0.18 (0, 4.65)
				CD	3.83 (0.42, 31.95)	2.9 (0.22, 47.38)
					FVBG	0.76 (0.03, 27.1
						PRP

*ABG* autologous bone grafting, *ABG* + *BMAC* autologous bone grafting and bone marrow aspirate concentrate, *BMAC* bone marrow aspirate concentration, *CD* core decompression, *FVBG* free vascular vascularized bone grafting, *MSCs* mesenchymal stem cells, *PRP* platelet-rich plasma

**Table 6:** The results of node-splitting method for ONFH progression

Comparison	ONFH progression		*P* value
CD vs. ABG	Direct	2.7 (0.13, 5.9)	0.37
	Indirect	2.2 (0.3, 3.0)	
FVBG vs. ABG	Direct	1.0 (0.09, 8.9)	0.37
	Indirect	0.1 (0.01, 14)	
MSCs vs. BMAC	Direct	0.3 (0.01, 9.2)	0.88
	Indirect	0.4 (0.01, 23)	
CD vs. BMAC	Direct	4.0 (1.3, 19)	0.89
	Indirect	2.9 (0.02, 4.2)	
CD vs. MSCs	Direct	9.1 (0.26, 46)	0.88
	Indirect	13 (0.39, 61)	
FVBG vs CD	Direct	0.04 (0.01, 1.6)	0.36
	Indirect	0.36 (0.01, 1.5)	

*ABG* autologous bone grafting, *BMAC* bone marrow aspirate concentration, *CD* core decompression, *FVBG* free vascular vascularized bone grafting, *MSCs* mesenchymal stem cells, *MSCs* mesenchymal stem cells, *ONFH* osteonecrosis of the femoral head

**Table 7:** The results of node-splitting method for conversion to THA

Comparison	Conversion to THA		*P* value
CD vs. ABG	Direct	2.0 (0.37, 11)	0.75
	Indirect	1.1 (0.04, 41)	
FVBG vs. ABG	Direct	0.4 (0.09, 1.7)	0.74
	Indirect	0.8 (0.01, 13)	
MSCs vs. BMAC	Direct	0.3 (0.04, 2)	0.90
	Indirect	0.3 (0.01, 3.4)	
CD vs. BMAC	Direct	2.4 (1.1, 5.1)	0.88
	Indirect	3.0 (0.12, 17)	
CD vs. MSCs	Direct	9.1 (0.77, 35)	0.90
	Indirect	7.1 (1, 64)	
FVBG vs CD	Direct	0.4 (0.01, 7.1)	0.73
	Indirect	0.2 (0.02, 1.9)	

*ABG* autologous bone grafting, *BMAC* bone marrow aspirate concentration, *CD* core decompression, *FVBG* free vascular vascularized bone grafting, *MSCs* mesenchymal stem cells, *ONFH* osteonecrosis of the femoral head, *THA* total hip arthroplasty

### Femoral head necrosis progress

**Fig. 6:**
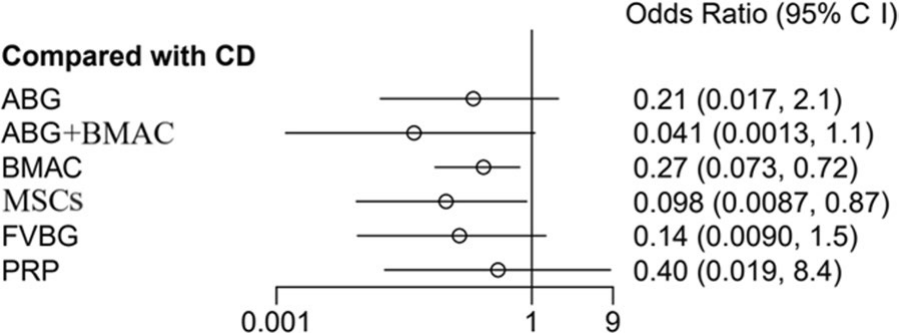
Forest plots of osteonecrosis of the femoral head (ONFH) progression. ABG: autologous bone grafting; ABG + BMAC: autologous bone grafting and bone marrow aspirate concentrate; BMAC: bone marrow aspirate concentration; CD: core decompression; CI, confidence interval; FVBG: free vascular vascularized bone grafting; MSCs: mesenchymal stem cells; PRP: platelet-rich plasma

All 17 articles reported ONFH progression. The NMA results showed that MSCs (OR: 0.098, 95% CI: 0.0087–0.87, SUCRA = 0.705) were the first effective technique for preventing ONFH progression, followed by BMAC (OR: 0.27, 95% CI: 0.073–0.73, SUCRA = 0.431). However, no significant difference was found among VBG, PRP, ABG + BMAC, ABG, or CD in preventing ONFH progression (*P* > 0.05) (Fig. 6).

### Conversion to total hip arthroplasty

**Fig. 7:**
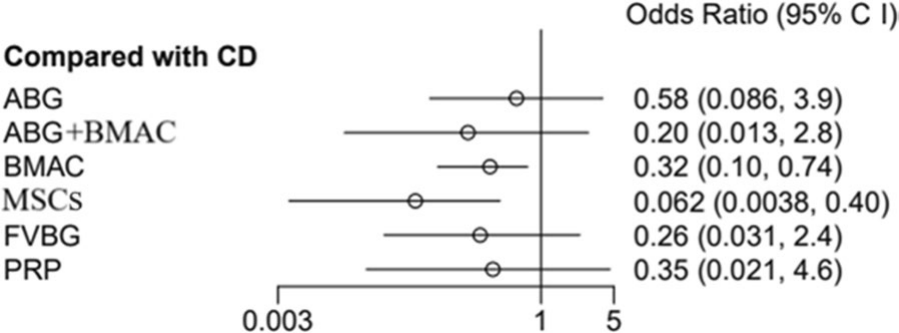
Forest plots of conversion to total hip arthroplasty (THA). ABG: autologous bone grafting; ABG + BMAC: autologous bone grafting and bone marrow aspirate concentrate; BMAC: bone marrow aspirate concentration; CD: core decompression; CI, confidence interval; FVBG: free vascular vascularized bone grafting; MSCs: mesenchymal stem cells; PRP: platelet-rich plasma

A total of 16 articles reported ONFH conversion to THA. MSCs (OR: 0.062, 95% CI: 0.0038–0.40, SUCRA = 0.902) and BMAC (OR: 0.32, 95% CI: 0.1–0.074, SUCRA** = **0.511) were the first and second effective techniques, respectively, for preventing ONFH conversion to THA. No significant difference was found among VBG, PRP, ABG + BMAC, ABG, or CD in preventing ONFH conversion to THA (*P* > 0.05) (Fig. 7).

## Discussion

This NMA investigated regenerative therapy for nontraumatic femoral head necrosis and included data from 17 clinical trials, including 1019 hips assigned to 6 different treatment options. The quality of evidence was generally low or moderate risk of bias. Our NMA found that MSCs and BMAC can prevent ONFH progression and conversion to THA. When ranked according to the SUCRA value, MSCs were the first, followed by BMAC; therefore, we concluded that MSCs and BMAC transplantation may be necessary after CD. To our knowledge, this is the first NMA to compare these six regenerative therapies, and these findings provide evidence for regenerative therapy for ONFH.

Previous traditional meta-analyses, such as the study by Andriolo et al. [11], did not compare different regenerative therapies separately with CD but combined the data of different regenerative therapies, which may have led to biased results. Zhang et al. [49] found that the combination of bone marrow stem cells had better prognosis outcomes than CD alone, such as ONFH progression or Harris Hip Score. However, during their study search, they classified bone and PRP graftings as stem cells, which broadened the study’s scope but inevitably increased its bias. Our study categorized regenerative therapy into six categories, which improved the search accuracy and obtained reliable study results. Migliorini et al. [50] found that bone marrow-derived cells had a lower probability of THA than CD, whereas conventional meta-analyses only compared bone marrow-derived cells with CD alone and could not compare multiple regenerative therapies. Bayesian NMA was used to review the regenerative therapy for ONFH in the CD (control), ABG, ABG + BMAC, BMAC, FVBG, MSCs, and PRP groups. Based on our NMA SUCRA analysis, MSCs ranked as the first intervention among the six regenerative therapies for preventing ONFH progression and conversion to THA, presumably because they provide better repair capacity [17, 39, 47]. However, ABG, ABG + BMAC, FVBG, PRP, and CD showed no significant differences in preventing ONFH progression and conversion to THA.

We derive the rationale behind this conclusion from the premise that regeneration therapy operates on the ability of cells and molecules to induce and promote tissue repair of ONFH. For example, BMAC contains many growth factors and non-mesenchymal cells, including endothelial, hematopoietic, and inflammatory cells, in which growth factors can induce stem cells to migrate to the injured site [40–42]. Many hematopoietic stem cells can provide vascular support and drive MSCs toward osteogenic differentiation [43]. With the progress in BMAC research, researchers have found that the non-progenitor cell component of BMAC may negatively affect its regeneration characteristics, limiting BMAC’s repair ability [44]. Generally, MSCs account for only 0.001–0.01% of the number of nucleated cells in BMAC [45, 46]. Studies have shown that high concentrations of MSCs can promote cartilage healing more than low concentrations of MSCs without causing adverse reactions [47]. Additionally, MSCs can differentiate into several cell types (including fibroblasts, chondroblasts, and other forms of tissue regeneration cells), thereby promoting tissue repair [48].

Although our study is the first Bayesian NMA to compare traditional CD with other regenerative therapies, it has limitations. First, it included only 17 related articles; therefore, the scale of direct comparison was limited. For example, only 80 and 245 hips were in the MSCs and BMAC groups, respectively; therefore, a limited sample size may increase statistical dispersion. Second, the study’s sample size was not large enough, potentially reducing the credibility of the results. Moreover, including patients with different Association Research Circulation Osseous (ARCO) stages leads to heterogeneity, and the prognoses of patients with different stages may differ. Considering these limitations, we recommend caution in our conclusions. Therefore, future research studies should include larger sample sizes covering the various treatments and ARCO/Ficat stages.

## Conclusion

Our NMA found that MSCs and BMAC were effective in preventing ONFH progression and conversion to THA among the six regenerative therapies. MSCs ranked first, followed by BMAC according to the SUCRA value. Based on our NMA results, MSCs and BMAC after CD may be necessary to prevent ONFH progression and conversion to THA. Furthermore, these findings provide evidence for regenerative therapy for ONFH.

### Supplementary Information

**Additional file 1.** Search Terms.

## Data Availability

The dataset supporting the conclusions of this article is included within the article.

## Abbreviations

ABG: Autologous bone grafting
ABG + BMAC: Autologous bone grafting and bone marrow aspirate concentrate
ARCO: Association research circulation osseous
BMAC: Bone marrow aspirate concentration
CI: Confidence interval
CD: Core decompression
FVBG: Free vascular vascularized bone grafting
MD: Mean difference
NMA: Network meta-analysis
OR: Odds ratio
ONFH: Osteonecrosis of the femoral head
PRP: Platelet-rich plasma
RCT: Randomized controlled trial
SUCRA: Surface under the cumulative ranking
THA: Total hip arthroplasty
