# Supplementary material for: Regenerative therapies for femoral head necrosis in the past two decades: a systematic review and network meta-analysis
Source: Stem Cell Res Ther. 2024 Jan 25;15:21. doi: 10.1186/s13287-024-03635-1 (PMC10809486; doi:10.1186/s13287-024-03635-1)
Supplement: Supplementary file 1 — Additional file 1. Search Terms. [file 13287_2024_3635_MOESM1_ESM.docx]

Supplementary Appendix 1. Search Terms

**Pubmed (MEDLINE)**

#1: ((((((((((((((((("Osteonecrosis"[Mesh]) OR (Osteonecroses[Title/Abstract])) OR (Bone Necrosis[Title/Abstract])) OR (Bone Necroses[Title/Abstract])) OR (Necroses, Bone[Title/Abstract])) OR (Necrosis, Bone[Title/Abstract])) OR (Necrosis, Avascular, of Bone[Title/Abstract])) OR (Avascular Necrosis of Bone[Title/Abstract])) OR (Bone Avascular Necrosis[Title/Abstract])) OR (Kienbock Disease[Title/Abstract])) OR (Kienbock's Disease[Title/Abstract])) OR (Kienboeck Disease[Title/Abstract])) OR (Kienboeck's Disease[Title/Abstract])) OR (Kienboecks Disease[Title/Abstract])) OR (Necrosis, Aseptic, of Bone[Title/Abstract])) OR (Aseptic Necrosis of Bone[Title/Abstract])) OR (Bone Aseptic Necrosis[Title/Abstract])) AND (((((("Femur Head"[Mesh]) OR (Femur Heads[Title/Abstract])) OR (Head, Femur[Title/Abstract])) OR (Femoral Head[Title/Abstract])) OR (Femoral Heads[Title/Abstract])) OR (Head, Femoral[Title/Abstract])) **6300**

#2: regenerative therapies [Title/Abstract] **2114**

#3: ((("Stem Cells"[Mesh]) OR (Progenitor Cells[Title/Abstract])) OR (Mother Cells[Title/Abstract])) OR (Colony-Forming Unit[Title/Abstract]) **292672**

#4: "Bone Marrow"[Mesh] **72482**

#5: ((("Bone Transplantation"[Mesh]) OR (Grafting, Bone[Title/Abstract])) OR (Bone Grafting[Title/Abstract])) OR (Transplantation, Bone[Title/Abstract]) **40860**

#6: (("Platelet-Rich Plasma"[Mesh]) OR (Plasma, Platelet-Rich[Title/Abstract])) OR (Platelet Rich Plasma[Title/Abstract]) **15131**

#7: #2 OR #3 OR #4 OR #5 OR #6 **409187**

#8: #1 AND #7 **828**

**Cochrane Central Register of Controlled Trials (CENTRAL) in The Cochrane Library (Wiley)**

#1: MeSH descriptor: [Osteonecrosis] explode all trees **352**

#2: (osteonecroses):ti,ab,kw OR (avascular necrosis):ti,ab,kw OR (bone necroses):ti,ab,kw OR (bone avascular necrosis):ti,ab,kw OR (aseptic necrosis of bone):ti,ab,kw **499**

#3: #1 OR #2 **780**

#4: MeSH descriptor: [Femur Head] explode all trees **133**

#5: (femur heads):ti,ab,kw OR (femoral head):ti,ab,kw OR (femoral heads):ti,ab,kw **1445**

#6: #4 OR #5 **1458**

#7: #3 AND #6 **314**

#8: MeSH descriptor: [Stem Cells] explode all trees **1152**

#9: (progenitor cells):ti,ab,kw OR (mother cells):ti,ab,kw OR (colony forming unit):ti,ab,kw **3411**

#10: MeSH descriptor: [Bone Marrow] explode all trees **1433**

#11: MeSH descriptor: [Platelet-Rich Plasma] explode all trees **967**

#12: (thrombocyte rich plasma):ti,ab,kw OR (platelet rich plasma):ti,ab,kw **3218**

#13: MeSH descriptor: [Bone Transplantation] explode all trees **1289**

#14: (grafting, bone):ti,ab,kw OR (bone grafting):ti,ab,kw OR (transplantation, bone):ti,ab,kw **8295**

#15: (regenerative therapies):ti,ab 117

#16: #8 OR #9 OR #10 OR #11 OR #12 OR #13 OR #14 OR #15 **16424**

#16: #7 AND #16 **58**

**EMBASE (Ovid)**

#1: 'bone necrosis'/exp **46762**

#2: 'osteonecroses': ti,ab **128**

#3: 'avascular necrosis': ti,ab **9650**

#4: 'bone necroses': ti,ab **51**

#5: 'bone avascular necrosis': ti,ab **14**

#6: 'kienboeck disease': ti,ab **4**

#7: 'aseptic necrosis of bone': ti,ab **88**

#8: #1 OR #2 OR #3 OR #4 OR #5 OR #6 OR #7 **48933**

#9: 'femoral head'/exp **18117**

#10: 'femur heads': ti,ab **121**

#11: 'femoral head': ti,ab **22385**

#12: 'femoral heads': ti,ab **4532**

#13: #9 OR #10 OR #11 OR #12 **30655**

#14: #8 AND #13 **10565**

#15: 'stem cell'/exp **482285**

#16: 'progenitor cells': ti,ab **87157**

#17: 'mother cells':ti,ab **1307**

#18: 'colony forming unit':ti,ab **7286**

#19: 'bone marrow'/exp **291324**

#20: 'thrombocyte rich plasma'/exp **19823**

#21: 'plasma, platelet-rich':ti,ab **83**

#22: 'platelet rich plasma':ti,ab **17806**

#23: 'bone transplantation'/exp **64237**

#24: 'grafting, bone':ti,ab **130**

#25: 'bone grafting':ti,ab **12691**

#26: 'transplantation, bone':ti,ab **441**

#27: 'regenerative therapies':ti,ab **2637**

#28: #15 OR #16 OR #17 OR #18 OR #19 OR #20 OR #21 OR #22 OR #23 OR #24 OR #25 OR #26 OR #27 **802983**

#29: #14 AND #28 **1745**

#30: #29 AND (2003:py OR 2004:py OR 2005:py OR 2006:py OR 2007:py OR 2008:py OR 2009:py OR 2010:py OR 2011:py OR 2012:py OR 2013:py OR 2014:py OR 2015:py OR 2016:py OR 2017:py OR 2018:py OR 2019:py OR 2020:py OR 2021:py OR 2022:py OR 2023:py) AND 'article'/it **1074**

#31: #30 AND 'human'/de **803**

#32: #31 AND [embase]/lim **663**

**Web of Science**

#1: (((((TS=(osteonecroses*)) OR TS=(avascular necrosis*)) OR TS=(bone necroses*)) OR TS=(bone avascular necrosis*)) OR TS=(kienboeck disease*)) OR TS=(aseptic necrosis of bone*) **10034**

#2: (((TS=(femoral head)) OR TS=(femur heads)) OR TS=(femoral head)) OR TS=(femoral heads) **24584**

#3: #2 AND #1 **4619**

#4: (((TS=(stem cell)) OR TS=(progenitor cells)) OR TS=(mother cells)) OR TS=(colony forming unit) **721234**

#5: ((TS=(thrombocyte rich plasma)) OR TS=(plasma, platelet-rich)) OR TS=(platelet rich plasma) **18284**

#6 ((TS=(bone transplantation)) OR TS=(grafting, bone)) OR TS=(bone grafting) **157546**

#7 TS=(bone marrow) **312411**

#8 TS=(regenerative therapies) **22259**

#10: #4 OR #5 OR #6 OR #7 OR #8 **972066**

#11: #3 AND #10 **1289**

#12: PY=(2003-2023) **52745977**

#13: #11 AND #12 **1042**
